# Abrogation of store-operated Ca^2+^ entry protects against crystal-induced ER stress in human proximal tubular cells

**DOI:** 10.1038/s41420-019-0203-5

**Published:** 2019-08-05

**Authors:** Farai C. Gombedza, Samuel Shin, Yianni L. Kanaras, Bidhan C. Bandyopadhyay

**Affiliations:** 1 Calcium Signaling Laboratory, Research Service, Veterans Affairs Medical Center, 50 Irving Street NW, Washington, DC 20422 USA; 2 Division of Renal Diseases & Hypertension, Department of Medicine, The George Washington University, 2150 Pennsylvania Avenue NW, Washington, DC 20037 USA; 3 Department of Biomedical Engineering, The Catholic University of America, 620 Michigan Avenue NE, Washington, DC 20064 USA

**Keywords:** Calcium signalling, Mechanisms of disease, Stress signalling

## Abstract

Calcium crystal internalization into proximal tubular (PT) cells results in acute kidney injury, nephrocalcinosis, chronic kidney disease (CKD), and kidney-stone formation. Ca^2+^ supersaturation in PT luminal fluid induces calcium crystal formation, leading to aberrant crystal internalization into PT cells. While such crystal internalization produces reactive oxygen species (ROS), cell membrane damage, and apoptosis; the upstream signaling events involving dysregulation of intracellular Ca^2+^ homeostasis and ER stress, remain largely unknown. We have recently described a transepithelial Ca^2+^ transport pathway regulated by receptor-operated Ca^2+^ entry (ROCE) in PT cells. Therefore, we examined the pathophysiological consequence of internalization of stone-forming calcium crystals such as calcium phosphate (CaP), calcium oxalate (CaOx), and CaP + CaOx (mixed) crystals on the regulation of intracellular Ca^2+^ signaling by measuring dynamic changes in Ca^2+^ transients in HK2, human PT cells, using pharmacological and siRNA inhibitors. The subsequent effect on ER stress was measured by changes in ER morphology, ER stress-related gene expression, endogenous ROS production, apoptosis, and necrosis. Interestingly, our data show that crystal internalization induced G-protein-coupled receptor-mediated sustained rise in intracellular Ca^2+^ concentration ([Ca^2+^]_i_) via store-operated Ca^2+^ entry (SOCE); suggesting that the mode of Ca^2+^ entry switches from ROCE to SOCE following crystal internalization. We found that SOCE components—stromal interacting molecules 1 and 2 (STIM1, STIM2) and ORAI3 (SOCE) channel were upregulated in these crystal-internalized cells, which induced ER stress, ROS production, and cell death. Finally, silencing those SOCE genes protected crystal-internalized cells from prolonged [Ca^2+^]_i_ rise and ER stress. Our data provide insight into the molecular mechanism of crystal-induced Ca^2+^ dysregulation, ER stress, and PT cell death and thus could have a translational role in treating crystal nephropathies including kidney stones. Taken together, modulation of Ca^2+^ signaling can be used as a tool to reverse the pathological consequence of crystal-induced conditions including cardiovascular calcification.

## Introduction

Crystals are often present in the renal tubular fluid of both stone-forming and nonstone-forming individuals^1^, inhibitors of crystallization rapidly cover such crystals following their formation, which facilitates their excretion^2^. However, such elimination processes could be hindered, when the crystals adhere to the tubular lining^3^. Interestingly, several studies have proposed that the onset of nephrolithiasis lies within the nephron, moreso, renal biopsies of some stone-formers presented with crystals inside renal tubular cells^4,5^. Thereafter, many in vitro studies have shown that calcium crystals, such as CaOx monohydrate, are taken up by renal tubular cells and subsequently influence the process of kidney-stone formation^6^. The proximal tubular (PT) cells are more prone to crystal internalization than distal tubular (DT) cells, due to their greater crystal binding affinity^7^. Interestingly, PT cells are also much more susceptible to insult/injury from extracellular stimuli^8^. Also, crystal size and shape determine the extent of internalization in various tubular cell types^9^. Internalization of CaP and CaOx crystals into PT cells has been shown to induce upregulation of inflammatory mediators, cellular damage, apoptosis, and renal interstitial fibrosis^10^. However, the effect of mixed (CaP + CaOx) crystals, which are usually the most abundant in calcium nephrolithiasis, has not yet been studied. Further, this crystal-induced cytotoxicity as a downstream mechanism toward cell death has been extensively studied^6,11–13^, which could be linked to the upstream mechanisms involving endoplasmic reticular (ER) and plasma membrane (PM) signaling in crystal-induced condition.

It is well understood that ER–PM Ca^2+^ signaling is critically involved in a variety of deleterious physiological responses^14,15^. Moreover, dysregulation of intracellular Ca^2+^ concentration [(Ca^2+^)_i_] due to prolonged Ca^2+^ influx can elicit reactive oxygen species (ROS) dependent proapoptotic signals^16^. Furthermore, preformed CaP crystals induced ROS and subsequent cell damage, which results in the aggregation of calcium crystals^17^. Several studies show that crystal-induced effects promote inflammatory and apoptotic responses^10^. However, the ER-functional status and upstream PM pathway of Ca^2+^ signaling resulting from crystal internalization into the cells are unknown. Store-operated Ca^2+^ entry (SOCE), an ER-dependent Ca^2+^ entry mechanism, which is stimulated by a reduction in Ca^2+^ levels in the ER, has been shown to control ER-dependent downstream signaling effect^18^. SOCE serves as a major mechanism for triggering Ca^2+^ influx into the non‐excitable cells, which helps maintain [Ca^2+^]_i_, and to prevent any downstream deleterious event. SOCE comprises of two Ca^2+^ sensors STIM1/2 located on the surface of the ER and three structurally related pore‐forming subunits (ORAI1/2/3), CRAC channels, located in the PM^19,20^. However, we do not know whether the internalization of crystals can have consequence on the ER–PM Ca^2+^ signaling mechanism in PT cells. Therefore, we focused on the mechanism of SOCE-mediated Ca^2+^ signaling events in CaOx, CaP, and mixed crystal-induced human PT cells (HK2). We show here that the crystal internalization into PT cells results in a sustained [Ca^2+^]_i_ rise via a STIM–ORAI3 SOCE-mediated mechanism, which drives the downstream ER stress response, ROS-induced cell damage, and apoptosis in HK2 cells. Blockade of such SOCE-mediated mechanism alleviates the deleterious effect of crystal internalization.

## Results

### Crystal internalization elicits prolonged [Ca^2+^]_i_ rise

**Fig. 1:**
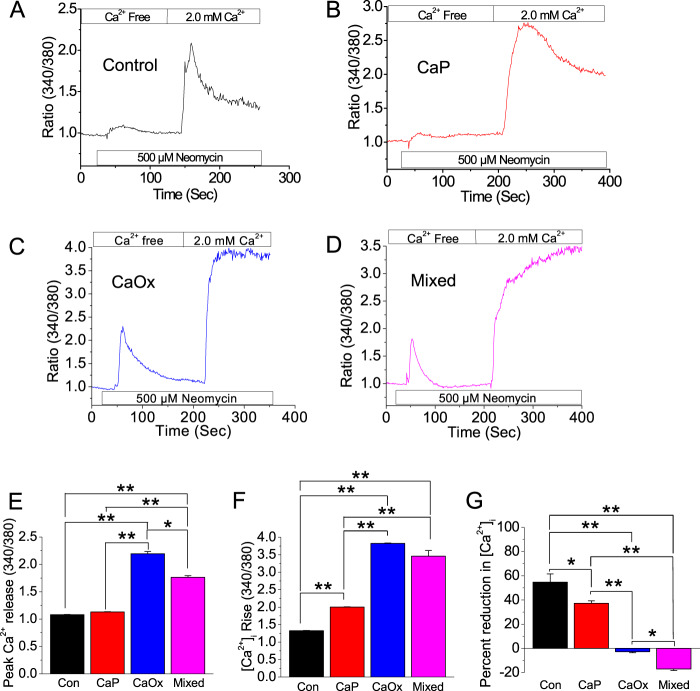
Crystal internalization induces sustained [Ca^2+^]_i_ rise in HK2 cells. Control (noncrystal) CaP, CaOx, and CaP + CaOx (mixed) crystals were introduced into HK2 cells for 24 h. Crystal internalization effects on [Ca^2+^]_i_ mobilization in GPCR-activated HK2 cells were measured by Ca^2+^ imaging. Mean fluorescent traces of Fura-2 loaded HK2 cells bathed in Ca^2+^-free solution and then 2.0 mM Ca^2+^ and neomycin CaSR activation were obtained for **a** Control; **b** CaP; **c** CaOx; and **d** Mixed crystals. Representative bar diagrams depict (**e**) peak [Ca^2+^]_i_ release; **f** [Ca^2+^]_i_ rise; and **g** percent reduction Ca^2+^ entry. Two-tailed t-test was used for statistical comparison in (**e**–**g**). Statistically significant differences are indicated (mean ± SEM) from four different experiments. Levels of significance are indicated as *p < 0.05; **p < 0.01 as shown in the bar diagrams

Crystal internalization into PT cells induces cytotoxicity, however, it is unknown, if such effect is linked to any dysregulation in ER–PM Ca^2+^ signaling mechanism. Thus, to examine such Ca^2+^ signaling, we tested the Ca^2+^-sensing receptor (CaSR) as G-protein-coupled receptor (GPCR)-mediated mechanism upon internalization of CaP, CaOx, and mixed crystals. We found that crystal internalization elicited an increase in Ca^2+^ release and produced a greater response in the Ca^2+^ rise due to Ca^2+^ entry compared with the noninternalized cells (Fig. 1a–d). CaP, CaOx, and mixed crystals internalizations were confirmed by differential (pH 4.3 & 6.8) AR staining (Supplementary Fig. 1A). Interestingly, Ca^2+^ responses among different crystal-internalized conditions were distinct; CaOx crystals induced greater Ca^2+^ release compared with CaP and mixed crystals (Fig. 1e). Furthermore, while CaSR-activated cells with CaOx crystals elicited greater [Ca^2+^]_i_ rise compared with those with CaP and mixed crystals (Fig. 1f), cells with mixed crystals displayed a further continuous rise in [Ca^2+^]_i_ compared with CaP and CaOx (Fig. 1g), suggesting that mixed crystals more effectively dysregulate the ER–PM Ca^2+^ signaling mechanism in PT cells. Together, our results suggest that crystal internalization initiated a disruption in Ca^2+^ mobilization, which could lead to ER stress.

### CaP and/or CaOx crystal internalization elicits SOCE pathway in HK2 cells

**Fig. 2:**
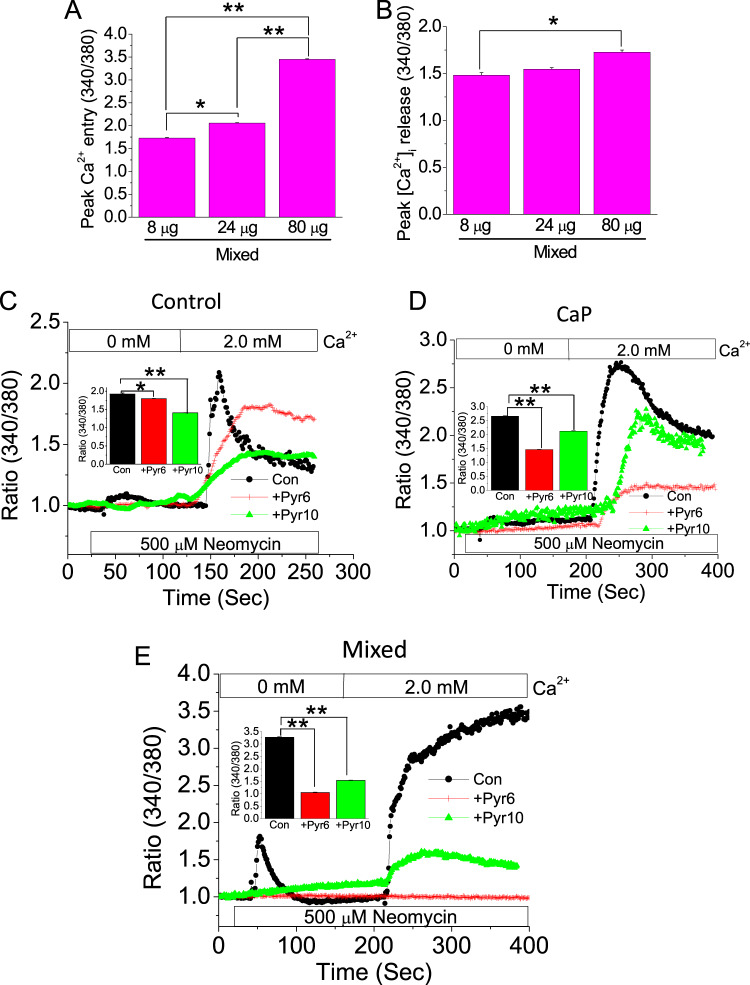
SOCE mediates Ca^2+^ entry in crystal-internalized HK2 cells. Mixed crystals were introduced into HK2 cells in a concentration-dependent manner (crystal concentrations: 8 µg/mL, 24 µg/mL, and 80 µg/mL) for 24 h. Following mixed crystal internalization, effects on [Ca^2+^]_i_ mobilization on CaSR-activated HK2 cells were measured by Ca^2+^ imaging. Representative bar diagrams depict **a** peak Ca^2+^ entry and, **b** peak [Ca^2+^]_i_ release. To delineate the pathway of Ca^2+^ entry, **c** control (noncrystal); **d** CaP; and **e** Mixed crystals were introduced into HK2 cells; following crystal internalization, cells were left untreated (control) or treated with SOCE blocker (Pyr6) or ROCE blocker (Pyr10). Mean fluorescent traces of Fura-2 loaded HK2 cells bathed in Ca^2+^-free solution and then 2.0 mM Ca^2+^ and neomycin CaSR activation were obtained for **c** Control; **d** CaP; and **e** Mixed crystals. Inset bar diagrams in **c**–**e** represent peak [Ca^2+^]_i_ rise. HK2 cells were incubated overnight with (**c**) control, CaP crystals (**d**), and mixed crystals (**e**). Cells were bathed in Ca^2+^-free solution and incubated for 5 minutes with 3 µM of Pyr6 or 10, and 50 µM neomycin was applied, followed by 2.0 mM Ca^2+^. Two-tailed *t*-test was used for statistical comparison in **a** and **b**. Statistically significant differences are indicated (mean ± SEM) from four different experiments. Levels of significance are indicated as *p < 0.05; **p < 0.01 as shown in the bar diagrams

Next, we examined if those crystal-induced conditions produced an exponential effect on ER–PM signaling upon mixed crystal internalization, since calcium kidney stones are primarily composed of a heterogeneous mixture of CaP + CaOx crystals. We tested whether such crystal internalization into PT cells could disrupt Ca^2+^ mobilization in a concentration-dependent manner. Our data shows that crystal internalization-induced [Ca^2+^]_i_ entry into HK2 cells is concentration-dependent (8, 24, and 80 µg/mL) manner in response to GPCR activation. Interestingly, [Ca^2+^]_i_ release was increased between 8 and 80 µg/mL but not between 24 and 80 µg/mL mixed crystals (Fig. 2a, b). This differential effect could be due to a greater ER stress resulting from internalization of higher concentrations of mixed crystal. Since we observed dysregulated GPCR-mediated Ca^2+^ entry following crystal internalization, we next sought to delineate the downstream pathway of Ca^2+^ entry elicited by crystal internalization. Since we used SOCE blocker, Pyr6, and ROCE (receptor-operated Ca^2+^ entry) blocker, Pyr10 in our previous study to differentiate between the role SOCE vs. ROCE, respectively^21,22^, we introduced CaP, or mixed crystals (8 µg/mL) into HK2 cells, treated cells with Pyr6 (3 µM) or Pyr10 (3 µM) and performed Ca^2+^ imaging experiments to measure Ca^2+^ entry induced by neomycin. Our data show that Pyr10 significantly inhibited the [Ca^2+^]_i_ rise in control cells induced by CaSR activation, indicating that the main Ca^2+^ signaling pathway under physiological condition is the ROCE. In contrast, Pyr6 decreased [Ca^2+^]_i_ rise in crystal-internalized cells to a greater extent than Pyr10 (Fig. 2c–e), suggesting that the mode of Ca^2+^ entry switches from ROCE to SOCE upon crystal internalization. Thus SOCE serves as the primary mediator of Ca^2+^ mobilization in these crystal-internalized PT.

### Increased Ca^2+^ entry in SOCE activated cells with CaP, CaOx, or mixed crystals

**Fig. 3:**
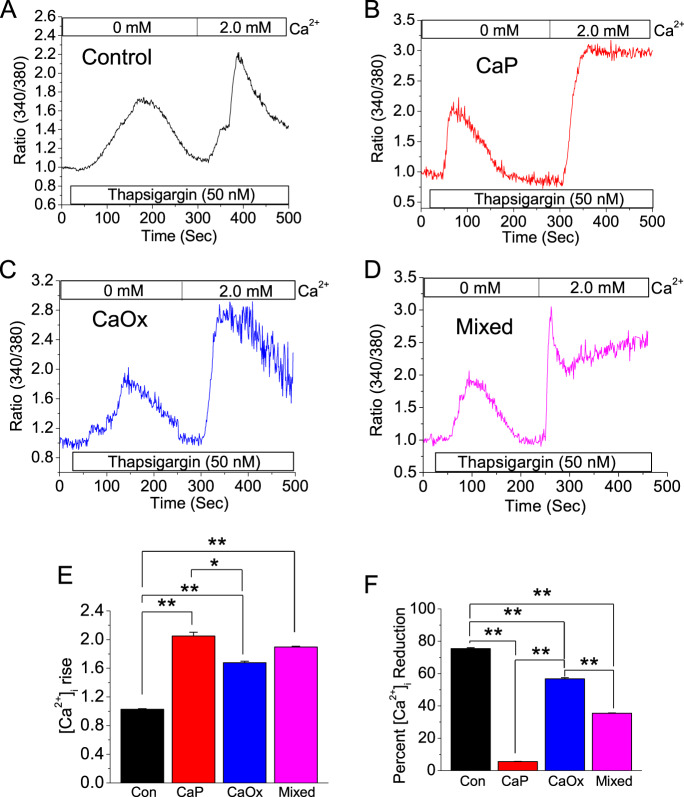
SOCE activation elevates Ca^2+^ entry crystal-internalized HK2 cells. Control (noncrystal) CaP, CaOx, and CaP + CaOx (mixed) crystals were introduced into HK2 cells for 24 h. Effects of tharpsigargin-induced SOCE activation on Ca^2+^ mobilization in crystal-internalized HK2 cells were measured by Ca^2+^ imaging. Mean fluorescent traces of Fura-2 loaded HK2 cells bathed in Ca^2+^-free solution and then 2.0 mM Ca^2+^ and tharpsigargin-induced SOCE activation were obtained for **a** Control; **b** CaP; **c** CaOx; and **d** Mixed crystals. Representative bar diagrams depict **e** [Ca^2+^]_i_ rise and **f** percent reduction Ca^2+^ entry. Two-tailed *t*-test was used for statistical comparison in **e** and **f**. Statistically significant differences are indicated (mean ± SEM) from four different experiments. Levels of significance are indicated as *p < 0.05; **p < 0.01 as shown in the bar diagrams

Since we found that the SOCE is the major Ca^2+^ entry pathway in crystal-internalized HK2 cells, we performed Ca^2+^ imaging experiments. Here we activated SOCE by thapsigargin (Tg; SERCA pump inhibitor is known to activate SOCE upon ER-Ca^2+^ stores depletion)^23^. To further investigate the difference in dynamics of Ca^2+^ mobilization among HK2 cells internalized with CaP, CaOx, and mixed crystals. We found that crystal-internalized cells demonstrated a greater [Ca^2+^]_i_ rise compared with the noninternalized (control) HK2 cells (Fig. 3a–e), indicating an upregulation of SOCE in these cells. Interestingly, while CaP crystal-internalized cells presented the least amount of [Ca^2+^]_i_ reduction (Fig. 3b, f), the cells with internalized mixed crystals resulted in less [Ca^2+^]_i_ reduction, but also with an inflection of its Ca^2+^ entry, leading to greater [Ca^2+^] entry (Fig. 3d, f), showing that the mixed crystals have greater potential in leading to ER stress through SOCE.

### Crystal internalization induces endoplasmic reticulum stress in human PT cells

**Fig. 4:**
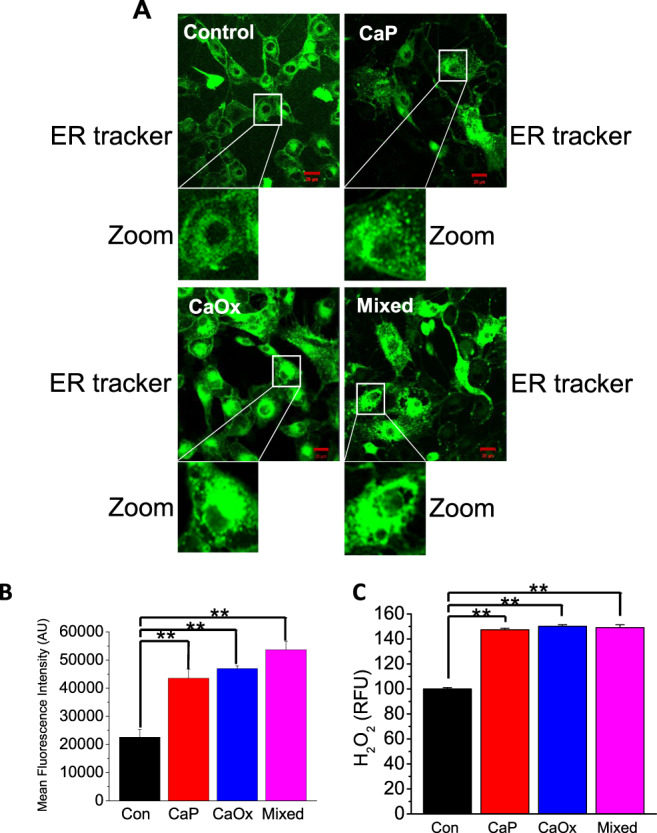
Crystal internalization alters ER morphology in HK2 cells. Control (noncrystal) CaP, CaOx, and CaP + CaOx (mixed) crystals were introduced into HK2 cells for 24 h. To determine ER subcellular localization and morphology, cells were stained with fluorescent ER tracker (green) and live-cell imaging was performed by confocal microscopy. **a** Representative ER staining of Control; CaP; CaOx; and Mixed crystal conditions were obtained. Images were collected with a 63× objective. **b** Mean fluorescence intensity was measured in six microscopic fields and quantified, intensity was quantified using ImageJ software version 1.52a (NIH); *n* = 6. **c** ER stress is associated with oxidative stress, ROS release was measured with ROS measurement kit following CaP, CaOx, and mixed crystal internalization for 24 h. Statistically significant differences are indicated (mean ± SEM). Experiments were performed in triplicates. Two-tailed *t*-test was used for statistical comparison in **b** and **c**. Levels of significance are indicated as *p < 0.05; **p < 0.01 as shown in the bar diagrams

ER Ca^2+^ levels are tightly monitored and maintained, we have shown previously that excess [Ca^2+^]_i_ is involved in the modulation of apoptosis^15^, however, we do not know the role of ER–PM Ca^2+^ signaling in this process. Since we found that CaP, CaOx, and mixed crystal internalization induces sustained Ca^2+^ entry and aberrant Ca^2+^ release from ER, we sought to understand the effects of crystal internalization on ER stress. We examined the subcellular localization and morphology of ER by performing live-cell imaging on HK2 cells with ER tracker (ER-specific marker; green) following internalization of CaP, CaOx, and mixed crystals. Similar to previous findings that ER stress induces dysregulation of ER morphology^24^, we found that crystal internalization induced swelling of the ER and formation of ER tracker positive vesicle-like ER structures (Fig. 4a). We quantified fluorescence intensity and showed that crystal internalization induced upregulation of ER-positive fluorescent stain, indicating ER stress (Fig. 4b). ER stress and impaired Ca^2+^ mobilization are both associated with increased mitochondrial ROS production. To examine the effect of crystal internalization on ROS production, we measured ROS release following CaP, CaOx, and mixed crystal internalization. Significantly, we found that crystal internalization remarkably increased ROS production (Fig. 4c). Taken together, our findings suggest that crystal-internalization induced ER stress-linked to ROS production and impaired cellular homeostasis, which could induce downstream apoptotic processes.

### Crystal internalization induces PM damage and apoptosis in HK2 cells

**Fig. 5:**
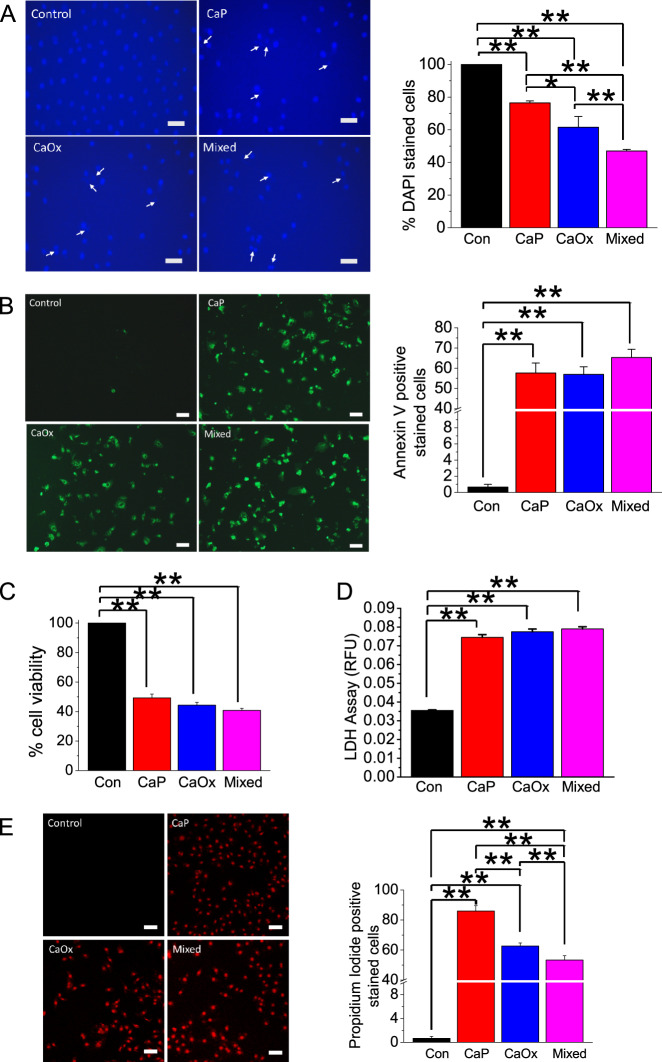
Crystal internalization induces LDH release and apoptosis in HK2 cells. Control (noncrystal) CaP, CaOx, and CaP + CaOx (mixed) crystals were introduced into HK2 cells for 24 h. Cell death was determined by **a** DAPI staining and **b** Annexin V-labeling. **c** Cell viability was determined relative to control (100%) by methylene blue staining. **d** LDH release was determined by measuring conversion of purple tetrazolium salt into red formazan. **e** Necrosis was detected with PI staining. Statistically significant differences are indicated (mean ± SEM). Experiments were performed in triplicates. Two-tailed *t*-test was used for statistical comparison. Levels of significance are indicated as *p < 0.05; **p < 0.01 as shown in the bar diagrams. Scale bar, 100 µm

Since crystal internalization induced overproduction of ROS, we next evaluated the effect of crystal internalization on cellular damage and cytotoxicity in HK2 cells. We found elevated crystal-induced apoptotic responses using DAPI staining (Fig. 5a) and Alexa Fluor 488-labeled annexin-V (Fig. 5b). Similarly, cell viability also decreased due to crystal internalization indicating apoptosis (Fig. 5c). Further, lactate dehydrogenase (LDH) release, a measure of PM damage, and PI labeling, a measure of necrosis; were increased following crystal internalization (Fig. 5d, e). Thus, our results indicate that crystal internalization resulted in cellular damage, necrosis, and cellular death simultaneously with abnormal ER–PM Ca^2+^ signaling, which are likely due to overproduction of ROS and impaired cellular homeostasis.

### Crystal internalization drives the expression and function of SOCE mediators STIM and ORAI

**Fig. 6:**
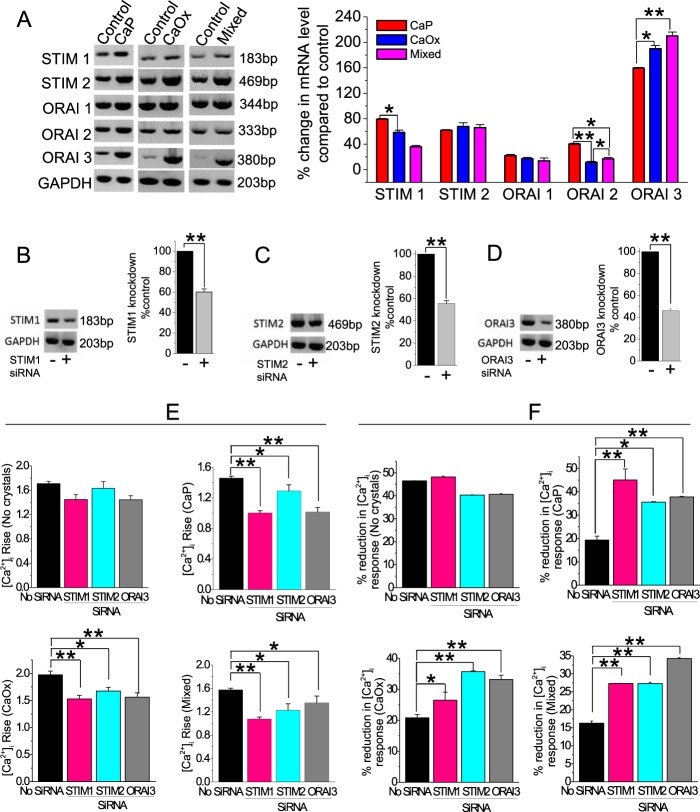
SOCE mediates crystal induced Ca^2+^ mobilization. Crystal internalization induces upregulation of STIM and ORAI channel expression. Control (noncrystal) CaP, CaOx, and CaP + CaOx (mixed) crystals were introduced into HK2 cells for 24 h. **a** mRNA expression levels of STIM1, 2, and ORAI channels were analyzed by PCR. ORAI and STIM expression is mediated by SOCE in HK2 cells. HK2 cells were incubated and representative blots were obtained, relative mRNA expression was quantified and represented as bar diagram. **b** To confirm knockdown following siRNA (10 nM), **b** STIM1; **c** STIM2; and **d** ORAI3 transcript were determined by PCR, representative blots were obtained and quantified as bar diagrams. **e** To ascertain the role of STIM12 and ORAI3 channel in crystal internalization, STIM1, 2, and ORAI3 expression was knocked down using siRNA, cells were then introduced with or without CaP, CaOx, and mixed (CaP + CaOx) crystals for 24 h. Following crystal internalization, cells were loaded with fura-2AM and Ca^2+^ influx was determined and represented as **d** [Ca^2+^] rise and **e** Ca^2+^ influx was determined and represented as reduction in [Ca^2+^] response. Two-tailed *t*-test was used for statistical comparison. Statistically significant differences are indicated (mean ± SEM) from three different experiments. Levels of significance are indicated as *p < 0.05; **p < 0.01 as shown in the bar diagrams

Since we found that SOCE is the major Ca^2+^ entry pathway in these crystal-internalized cells, we measured the expression of SOCE components STIM1, 2, and ORAI channels 1, 2, and 3. Our results show that crystal internalization markedly increased the gene expression of STIM1, STIM2, and ORAI3 (Fig. 6a). We then knocked down STIM1, STIM2, and ORAI3 expression in HK2 cells using specific siRNA and measured Ca^2+^ entry into these crystal-internalized cells (Fig. 6b–d). Following knockdown with respective siRNA, we performed crystal internalization into these cells to examine the effect on Ca^2+^ release and entry to determine Ca^2+^ signaling profile. Our data show that STIM1, STIM2, and ORAI3 siRNA transfected cells decreased [Ca^2+^]_i_ rise compared with control (scrambled siRNA transfected) cells for all CaP, CaOx, and mixed crystal-internalized cells, whereas noninternalized cells did not show such response (Fig. 6e). We also analyzed the Ca^2+^ entry component and found that the percent reduction in [Ca^2+^]_i_ response was increased to a greater extent in STIM1, STIM2, and ORAI3 siRNA transfected cells compared with control (scrambled siRNA transfected) cells (Fig. 6f). Remarkably, the Ca^2+^ response (rise and reduction) by siRNA inhibitors corresponded to the expression of SOCE components in crystal-internalized cells. Taken together, our results confirm that ORAI3 is the SOCE channel acting in combination with STIM1, STIM2 as ER sensors to drive Ca^2+^ response in crystal-internalized human PT cells.

### Inhibition of SOCE protects against crystal-induced ER stress

**Fig. 7:**
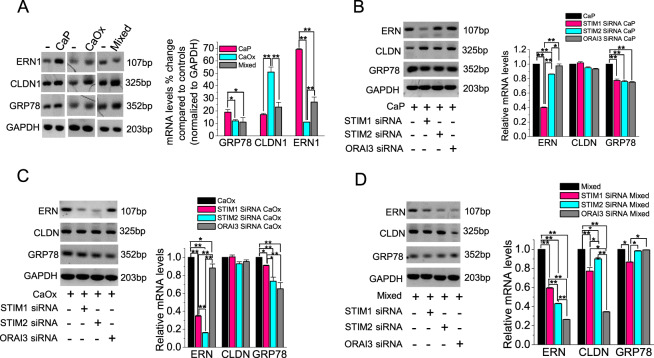
Inhibition of STIM1, STIM2, and ORAI3 channel alleviates ER stress. Control (noncrystal) CaP, CaOx, and CaP + CaOx (mixed) crystals were introduced into HK2 cells for 24 h. **a** mRNA levels of ER stress genes ERN (ERN1), CLDN (CLDN1), and GRP78 were analyzed by PCR and representative blots were obtained and quantified. STIM1, STIM2, and ORAI3 expression was knocked down using siRNA transfection (10 nM). Following transfection, **b** CaP; **c** CaOx, and **d** Mixed crystals were introduced into HK2 cells and mRNA levels of ER stress genes ERN1, CLDN1, and GRP78 were analyzed by PCR and representative blots were obtained and quantified. Two-tailed *t*-test was used for statistical comparison. Statistically significant differences are indicated (mean ± SEM) from three different experiments. Levels of significance are indicated as *p < 0.05; **p < 0.01 as shown in the bar diagrams

Our earlier findings suggested that crystal-internalization induced ER stress-linked with ROS production (Fig. 4). Thus, to investigate the effect of crystal internalization on ER stress, we measured the expression of ER stress genes such as the levels of ER stress-related to Nucleus signaling 1 (ERN1), claudin 1 (CLDN1), and Heat Shock Protein Family A (HSP70) Member 5 (GRP78) in PT cells. We mentioned earlier that crystal internalization induced sustained Ca^2+^ entry and altered ER morphology, both of which indicate ER stress. Here we show that crystal internalization upregulates the expression of ER stress-related genes compared with the noncrystal condition (Fig. 7a). We next examined the role of SOCE components on the expression of crystal-induced ER stress-related genes. Thus, we knocked down STIM1, STIM2, and ORAI3 expression using specific siRNAs, and then internalized the PT cells with crystals. Interestingly, we found that cells deficient in STIM1 were protected from ERN1 upregulation following CaP internalization (Fig. 7b). Significantly, cells deficient in STIM1 and STIM2 were also protected from ERN1 upregulation following CaOx internalization (Fig. 7c). Further, STIM1, STIM2, and ORAI3 deficient cells when internalized with mixed crystals, were shown to protect from upregulation of ERN1 expression in crystal-internalized condition, whereas protection of CLDN1 upregulation was found in ORAI3 deficient cells internalized with crystals (Fig. 7d). Thus, our results suggest that (i) inhibition of SOCE components that were upregulated due to crystal internalization in cells can protect against ER stress condition and (ii) the type (CaP/CaOx/mixed) of crystal internalization differentially regulate ER stress via SOCE components.

**Fig. 8:**
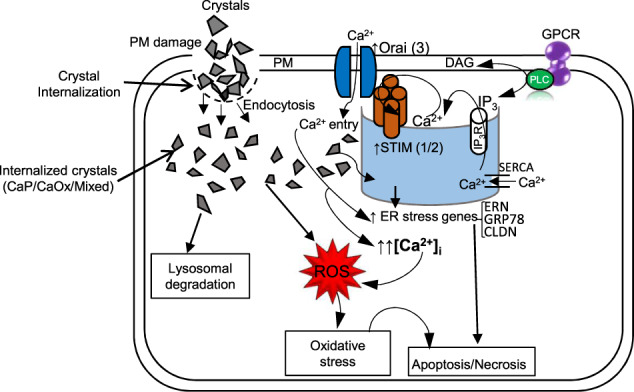
Schematic diagram of proposed mechanism of crystal-induced Ca^2+^ signaling regulating downstream effects. Crystal internalization via endocytosis elicits prolonged Ca^2+^ entry, ER stress responses, ROS production, oxidative stress, plasma membrane (PM) damage, and cell death in HK2 cells. ER stress responses include ER morphological changes and upregulation of ER stress genes. Crystal internalization promotes upregulation of SOCE components: STIM1/2 and ORAI3 which induces prolonged Ca^2+^ entry and leads to a continuous rise in [Ca^2+^]_i_, causing ROS generation, oxidative stress, and apoptosis/necrosis. CaOx Calcium Oxalate, CaP Calcium Phosphate, CaSR Calcium Sensing Receptor, CLDN Claudin; ER Endoplasmic Reticulum, ERN Endoplasmic Reticulum to Nucleus Signaling, GAPDH Glyceraldehyde 3-phosphate dehydrogenase, GPCR G-protein-coupled Receptor, GRP78 glucose-regulated protein, Mixed CaP + CaOx, PM Plasma Membrane, ROCE Receptor Operated Ca^2+^ Entry, ROS Reactive Oxygen Species, SOCE Store Operated Ca^2+^ Entry, STIM Stromal Interaction Molecule

## Discussion

In this study, we used kidney-stone-forming calcium crystals to understand the change in Ca^2+^ signaling status and the gene expression profile in those crystal-internalized cells, which could be used by these cells to ensure the adaptability to cope with the challenged cellular environments. Such changes incur the development of cellular stress and ROS generation; however, the mechanism of Ca^2+^ signaling in those crystal-internalized PT cells, which could be an important regulator driving the downstream events leading to fibrosis, inflammation, apoptosis, and/or necrosis is unknown. Our study for the first time demonstrated that the change in upstream Ca^2+^ signaling signature in human PT cells in crystal-internalized condition regulates the downstream effects (Fig. 8). Our data present a differential Ca^2+^ signaling response upon GPCR activation among the CaP, CaOx, or mixed crystal-internalized human PT cells. In fact, CaOx crystals induced a greater Ca^2+^ release compared with CaP and mixed crystals. An increase in the amplitude and duration of ER Ca^2+^ release has been shown as a marker for ER stress and of an ER/mitochondrial signaling pathway which are the beginning steps in inducing apoptosis^15^. Moreover, increased [Ca^2+^]_i_ rise through the mechanisms of Ca^2+^ signaling, has been shown to induce ER stress^25^, which may indicate the disruption of the Ca^2+^ signaling mechanism in CaOx and mixed crystal-internalized cells.

We performed crystal internalization at physiological pH to ensure the optimal operation of active transport mechanism involving actin cytoskeleton-mediated macropinocytosis^26^. Furthermore, we tested the Ca^2+^ influx as a measure of altered PM signaling function in these crystal-internalized cells. The amount of crystal was proportionate to the rise in [Ca^2+^]_i_, indicating that ROS production can be driven by crystal-internalized condition. While crystal–cell interaction has been marked as an essential element in the development of urinary stone disease^27^, it was reported as the earliest processes in the formation of kidney stones^28^. Therefore, the incremental effect on the Ca^2+^ signaling due to more crystal internalization is relevant as this effect can lead to greater ER stress. Some preclinical studies have provided evidence for crystal retention within the kidneys via attachment mainly to the brush border of proximal tubules in rodents. Therefore, our results using human PT cells could be more relevant toward exploring those mechanism of crystal-induced nephrolithiasis.

Our recent study in animal model show for the first time that PT cells are capable of transporting Ca^2+^ via a regulated ROCE pathway^21^. Surprisingly, our data show that as the crystals enters the PT cells, the mode of Ca^2+^ signaling switches from ROCE to SOCE, which may be needed to mediate in crystal-induced condition. Such Ca^2+^ signaling status in PT cells due to crystal internalization could be prompted to upregulate the deleterious effects such as enhanced ROS production, inflammatory mediators, cellular damage, apoptosis, and renal interstitial fibrosis^15^. Moreover, excess internal Ca^2+^ store depletion in epithelial cells can result in ER stress via SOCE Ca^2+^ pathway involving STIM protein^18^. Therefore, SOCE pathway due to crystal internalization, could determine the functional status in PT cells. In many cell types, SOCE has been described as proapoptotic pathway^29,30^. Further, this change in mode of Ca^2+^ entry pathway could be broadly true for other calcium crystal pathologies such as vascular smooth muscle calcifications or breast tissue microcalcifications; exploration of which is beyond the scope of the present manuscript. Since we observed ER-store depletion, which resulted in sustained Ca^2+^ entry and [Ca^2+^]_i_ rise, it is not surprising that such process may be working toward the altered intracellular microenvironment. Nevertheless, we found that CaP may be more effective in sustained [Ca^2+^]_i_ than CaOx, effect of which was slightly blunted by mixed crystal. Interestingly, the resulting crystal-induced Ca^2+^ signaling signature i.e., prolonged Ca^2+^ entry and rise in [Ca^2+^]_i_, was surprisingly similar to our recent study inhibiting sodium-calcium exchanger in mice PT cells, suggesting that crystal internalization could be similar to the disruption of intracellular Ca^2+^ transport machinery. It is possible that the other Ca^2+^ signaling pathways and [Ca^2+^]_i_ pool may have been affected due to crystal internalization into PT cells, mechanism of which is beyond the scope of the present manuscript.

ER stress has been shown to induce swelling of ER cisternae in the cytoplasm to sac-like vesicles^31^. We found that crystal-internalized condition resulted in morphological alterations to ER, further suggesting that crystal induction into PT cells has unpropitious effects on PT cellular function. It is possible that such crystal-induced changes in ER can affect the downstream function of protein synthesis and degradation, details of which can be planned in our future studies. Introduction of crystals induces ROS generation with impaired cellular function and cytotoxicity^11^. Therefore, we confirmed that crystal internalization drove upregulation of intracellular H_2_O_2_ generation. Notably, ROS-damage enhances the adhesive capacity due to increased expression of crystal binding protein in kidney tubular cells^32^, suggesting that PT crystal internalization may promote intracellular H_2_O_2_ generation, accompanying oxidative damage. Compounding altered Ca^2+^ signaling mode, crystal-induced damage and LDH release were elicited followed by cytotoxicity, and DNA fragmentation resulting in crystal-induced cell death.

STIM proteins are ER intraluminal Ca^2+^ sensors bound to PM ORAI channels, which drive SOCE to maintain [Ca^2+^]_i_ and ER function. Moreover, blockade of ORAI channels has been shown to prevent the development of renal fibrosis in mice with chronic kidney disease (CKD)^33^. Furthermore, overexpression of ORAI1 has been shown in other kidney-related diabetic disease^34^. Since we observed that crystal internalization switches the mode of Ca^2+^ signaling from ROCE to SOCE, we examined the expression of STIM and ORAI and found that both STIM1, STIM2, and ORAI3 channel expression were upregulated following crystal internalization. These findings were significant because they are consistent with observed sustained Ca^2+^ entry and ER stress, revealing a link between Ca^2+^ supersaturation and dysregulated Ca^2+^ mobilization in human PT cells.

ER stress has a role in acute kidney injury, CKD, and renal fibrosis^35^, however, its involvement in kidney stones remains unclear. In our study, crystal introduction into human PT cells, upregulated the expression of ER stress genes—ERN1, GRP78, and CLDN1. Interestingly, knockdown of ER Ca^2+^ sensor STIM1, STIM2, and PM ORAI3 channel using specific siRNAs markedly attenuated crystal-induced expression of ER stress genes. Notably, our data provides further evidence that targeting ER–PM Ca^2+^ regulation may provide a novel avenue for the development of therapeutic agents not only for kidney-stone disease, but also for other crystal-related nephropathies. Of note, our in vitro inhibition of STIM and ORAI using siRNA may have transient effects, which should be confirmed in PT-specific conditional knockout murine models to understand the role of these players in crystal-related pathologies. Crystal deposition resulting from luminal supersaturation of Ca^2+^, is a mainstay of renal disease such as nephrocalcinosis, oxalosis, and CKD. Crystal-internalization induced ER morphological alteration and promoted ER stress-induced gene upregulation, evidence of ER stress. Inhibition of STIM-gated SOCE alleviated crystal-induced ER stress, indicating that inhibition of SOCE components may protect the kidney from diseases involving aberrant crystal deposition. Our study provides clues for the mechanism of cell death by dysregulated Ca^2+^ signaling mediated by crystal internalization. Significantly, the current work highlights a mechanism for the deleterious effects of crystal internalization in human PT cells and provides evidence for targeting SOCE as a therapeutic option for the prevention of kidney diseases such as acute kidney injury, CKD, and renal fibrosis.

## Methods

### Reagents and chemicals

Calcium chloride, monosodium phosphate, disodium phosphate, sodium oxalate, tharpsigargin, Pyr6, Pyr10, neomycin, and other chemicals were purchased from Sigma-Aldrich (St. Louis, MO). Fura-2AM was purchased from Invitrogen (Carlsbad, CA). ER tracker was purchased from Cell Signaling Technology (Danvers, MA).

### Cell culture

Dulbecco’s modified Eagle’s medium (DMEM), fetal bovine serum (FBS), antibiotics (penicillin and streptomycin), and glutamine were purchased from Invitrogen. Human kidney proximal tubule epithelial cells (HK2) were obtained from Lonza (Walkersville, MD). HK2 cells were cultured in DMEM supplemented with 10% fetal bovine serum, 2 mM glutamine, and 1% penicillin/streptomycin at 37 °C in 5% CO_2_.

### Preparation of crystals

CaP, CaOx, and mixed crystals were prepared as described previously^6^. Briefly, CaP crystals were prepared by mixing solutions of 2.4 mM CaCl_2_, 0.9 mM Na_2_HPO_4_, and 5.8 mM NaH_2_PO_4_. CaOx crystals were prepared by mixing solutions of 2.4 mM CaCl_2_, 1.0 mM Na_2_C_2_O_4_. Mixed crystals were prepared by combining CaP and CaOx solutions. Crystals were prepared in HBSS to a concentration of 800 µg/ml. Mixed solutions were agitated for 30 min at RT and then centrifuged for 5 min (10,000 rpm), the supernatant was discarded, and the crystals were washed with HBSS twice.

### Crystal internalization

HK2 cells were seeded and grown to 80% confluency in complete media. For crystal induction, appropriate crystals (CaP, CaOx, or mixed crystals) (800 µg/ml) were added in serum-free media and incubated for 4 h at 37 °C in 5% CO_2_. After 4 h incubation, complete media (DMEM) with 10% FBS was added, and cells were incubated for a further 20 h.

### Alizarin Red staining

Alizarin red (3,4-Dihydroxy-9, 10-dioxo-2-anthracenesulfonic acid sodium) staining was performed as previously described to detect the presence of CaP and/or CaOx crystals^6^. Briefly, following crystal induction, cells were rinsed with HBSS without phenol red, Ca^2+^, and Mg^2+^, fixed with 3% paraformaldehyde for 10 min at RT, then washed with HBSS three times. Crystals/cells were then incubated in 2% alizarin red solution (pH = 4.3 or 6.8) and incubated at 37 °C for 15 min. Stained images were obtained using Zeiss Axiovision microscope (Supplemental Fig. 1A).

### siRNA transfection

HK2 cells were transfected with (10 nM) siRNA against STIM1 (Santa Cruz Biotechnology, Santa Cruz CA; sc-76589), STIM2 (Santa Cruz sc-76591), ORAI3 (Santa Cruz sc-76005), and scrambled siRNA-A (Santa Cruz sc-37007) as negative control. Transfection was performed using Lipofectamine-2000 Reagent (Thermo Fisher Scientific, Waltham, MA) according to manufacturer’s instructions.

### Fura-2 loading and measurement of intracellular [Ca^2+^]

Ratiometric (340/380) measurements of [Ca^2+^]_i_ were performed as described previously^15,21^. Briefly, Fura-2-loaded cells were placed on an IX81 motorized inverted microscope equipped with a IX2-UCB control box (Olympus USA, Center Valley, PA). For time-lapse fluorescence/ratiometric measurements, the IX81 microscope images were fed into a C9100-02 electron multiplier CCD camera with an AC adaptor A3472-07 (Hamamatsu, Bridgewater, NJ). A Lambda-LS xenon arc lamp and 10–2 optical filter changer (Sutter Inst. Novato, CA) were used as an illuminator capable of light output from 340 and 380 nm to a cutoff of 700 nm. All experiments were conducted in a microincubator with a constant temperature set at 37 °C and a gas mixture of 95% O_2_ and 5% CO_2_. Cells were bathed in Ca^2+^-free SES during the experiment. Ratiometric measurements of [Ca^2+^]_i_ were obtained using digital microscopy imaging software (SlideBook version 5.0, 3i, Intelligent Imaging Innovations, Denver, CO). Fura-2 fluorescence was recorded at an emission peak absorbance at 500 nm wavelength with excitation peak absorbance that continuously shifted at wavelengths of 340 and 380 nm. Time lapse was set at 250–500-time points at 1 s intervals in 50–150 cells, selected as region of interest (background fluorescence automatically subtracted prior to 340/380 ratio calculation and graphing). Analysis was performed offline using Slidebook^TM^ software and further analyzed using statistical analysis by Origin 6.1.

### H_2_O_2_ release measurement

H_2_O_2_ release from cells following crystal induction was assessed using H_2_O_2_ Cell-Based Assay Kit (Cayman Chemicals, Ann Arbor, MI). Pre-assay and assay preparations were performed according to manufacturer’s instructions. Assay was performed in triplicate. H_2_O_2_ release was assessed by subtracting the background wavelength (540 nm) from the emission wavelength (590 nm).

### DAPI staining

Apoptotic nuclei were detected using DAPI. Cell culture and crystal induction followed the same procedure as described earlier^15^. Following crystal induction, cells were fixed with 3% paraformaldehyde, permeabilized with 0.01% Triton X-100 in 0.1% BSA for 2 min, washed with 1 × PBS once, and incubated with DAPI (1 μg/ml in 1 × PBS pH 7.4) in 500 µl for 10 min at room temperature in the dark. After staining, the DAPI solution was removed; cells were washed two times with 1 × PBS and visualized under fluorescence microscopy (Axiovision, Carl Zeiss).

### Cell viability assay

HK2 cells were seeded and grown to 80% confluency. Cells were induced with crystals as described previously^6^. Following crystal induction, cells were trypsinized and stained with toluidine blue stain. Live cells were counted using a hemocytometer and standard procedure, and cell viability was calculated relative to control (no crystal) condition. Cell counts were performed in triplicates.

### Annexin V/PI staining for apoptosis necrosis assay

Apoptotic and necrotic cells were assessed using Alexa Fluro 488 Annexin V/Dead Cell apoptosis kit (Thermo Fisher Scientific). Staining was performed according to manufacturer’s instructions.

### LDH release measurements

LDH release from crystal-induced HK2 cells was measured using Pierce LDH Cytotoxicity Assay kit (Thermo Fisher Scientific) according to manufacturer’s instructions. Briefly, following crystal induction, cell culture media was collected and transferred to a 96-well plate in triplicates. LDH release reactions were performed and assessed by the subtraction of the absorbance at 680 nm (background) from the absorbance at 490 nm.

### RNA extraction and PCR

Total RNA’s were isolated from HK2 cells using TRIzol as previously described^15^. Subsequently, DNase treatments were performed, and RNA concentrations were measured using nanodrop spectrophotometer. Afterward, a cDNA synthesis kit (Promega, Madison, WI) was used to reverse transcribe the RNA into the cDNA, which were amplified with gene specific primers (Supplementary Fig. 1B) purchased from Invitrogen and Integrated DNA Technologies (Coralville, IA) using the master mix PCR amplification reagent (Promega). With a T100 Thermocycler (Bio-Rad, Hercules, CA), the following PCR conditions were used: one initial cycle at 95 °C for 3 min; 30–35 cycles of denaturation at 95 °C for 30 s, annealing at 55 °C for 30 s, and elongation at 72 °C for 45 s; an additional 5 min at 72 °C; and a final hold at 4 °C.

### Statistical analysis

Experimental results are expressed as mean ± S.E.M. Statistical comparisons were performed using Student’s unpaired *t*-test (two-tailed), in Origin 6.1. Statistically significant comparisons were accepted at *P* value < 0.05.

## Supplementary information

Supplementary Figure 1

Supplemental Material File #1
