# Correction: Abrogation of store-operated Ca^2+^ entry protects against crystal-induced ER stress in human proximal tubular cells

**DOI:** 10.1038/s41420-021-00445-9

**Published:** 2021-04-06

**Authors:** Farai C. Gombedza, Samuel Shin, Yianni L. Kanaras, Bidhan C. Bandyopadhyay

**Affiliations:** 1 Calcium Signaling Laboratory, Research Service, Veterans Affairs Medical Center, 50 Irving Street NW, Washington, DC, 20422 USA; 2 Division of Renal Diseases & Hypertension, Department of Medicine, The George Washington University, 2150 Pennsylvania Avenue NW, Washington, DC, 20037 USA; 3 Department of Biomedical Engineering, The Catholic University of America, 620 Michigan Avenue NE, Washington, DC, 20064 USA

**Keywords:** Calcium signalling, Mechanisms of disease, Stress signalling

Correction to: *Cell Death Discovery*

10.1038/s41420-019-0203-5 published online 5 August 2019

Following publication, an error was noticed in Fig. 5A, and in the legend of Fig. 5. In Fig. 5A, the top right (‘CaP’) panel was accidentally duplicated with the bottom left (‘CaOx’) panel. The corrected figure has been provided below, and the authors have updated the associated histogram and statistics.

In the Fig. 5 legend (A–E), an error was noticed that may have caused confusion around the nature of the replicates. The results were obtained from three individual experiments, rather than from triplicates. The correct legend is provided below.

**Fig. 5:**
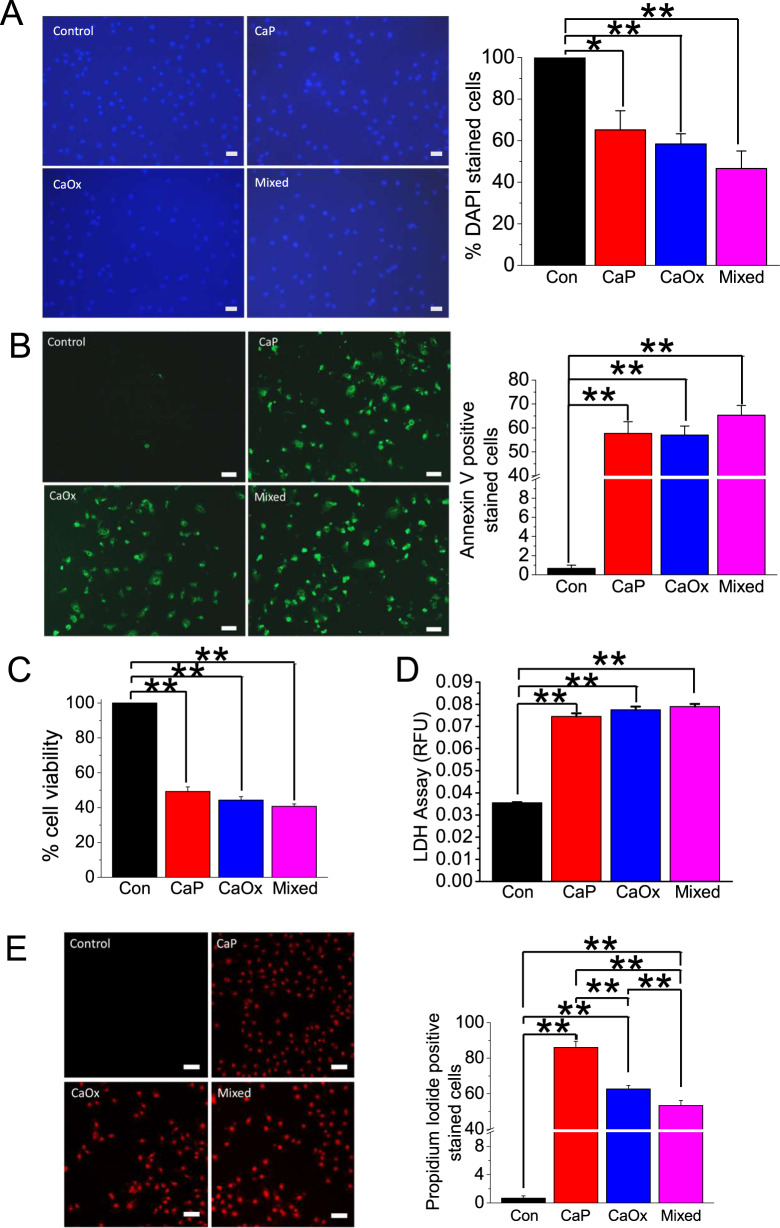
Crystal internalization induces LDH release and apoptosis in HK2 cells. Control (noncrystal) CaP, CaOx, and CaP + CaOx (mixed) crystals were introduced into HK2 cells for 24 h. Cell death was determined by **A** DAPI staining and **B** Annexin V labeling. **C** Cell viability was determined relative to control (100%) by methylene blue staining. **D** LDH release was determined by measuring conversion of purple tetrazolium salt into red formazan. **E** Necrosis was detected with PI staining. Statistically significant differences are indicated (mean ± SEM). Experiments were performed in triplicates. Data are presented from three individual experiments (*n* = 3). Two-tailed *t* test was used for statistical comparison. Levels of significance are indicated as *p < 0.05 and **p < 0.01 as shown in the bar diagrams. Scale bar, 100 µm.

The authors apologise for any inconvenience caused by these errors.

