# Supplementary material for: Abrogation of store-operated Ca2+ entry protects against crystal-induced ER stress in human proximal tubular cells
Source: Cell Death Discov. 2019 Aug 5;5:124. doi: 10.1038/s41420-019-0203-5 (PMC6680047; doi:10.1038/s41420-019-0203-5)
Supplement: Supplementary file 1 — Supplementary Figure 1 [file 41420_2019_203_MOESM1_ESM.pdf]

A

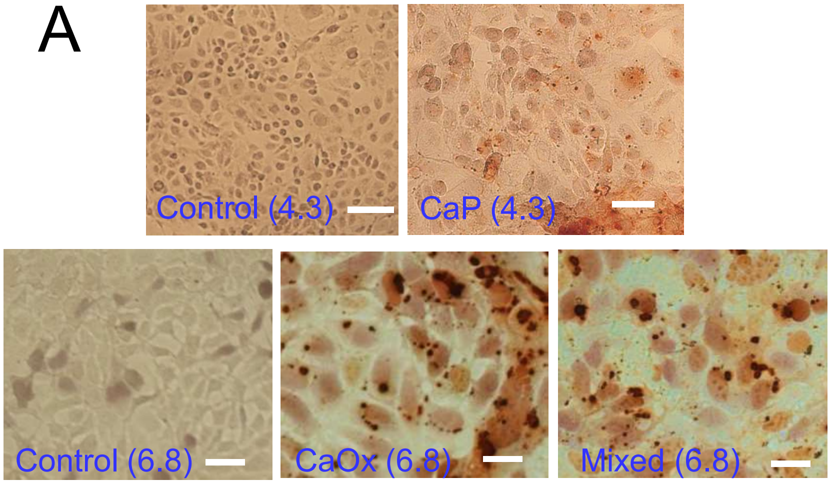

B

Primer	Sequence (sense, antisense)
hSTIM1	5'-TGT GGA GCT GCC TCA GTA TG -3'<br>5'-AAG AGA GGA GGC CCA AAG AG -3'
hSTIM2	5'-TGT CAC TGA GTC CAC CAT GC -3'<br>5'-GGG CGT GTT AGA GGT CCA AA -3'
hOrai1	5'-AGC AAC GTG CAC AAA TCT CAA -3'<br>5'-GTC TAT GGC TAA CCA GTG A -3'
hOrai2	5'-CGG CCA TAA GGG CAT GGA TT -3'<br>5'-TTG TGG ATG TTG CTC ACG GC -3'
hOrai3	5'-CTC TTC CTT GCT GAA GTT GT -3'<br>5'-CGA TTC AGT TCC TCT AGT TC -3'
hERN1	5'-AGA GAA GCA GAC TTT GTC -3'<br>5'-GTT TTG GTG TCG TAC ATG GTG A -3'
hCLDN1	5'-GGT GCA GAA GAT GAG GGC TG -3'<br>5'-AGC CAG TGA AGA GAG CCT GAC G -3'
hGRP78	5'-GGA TCA TCA ACG AGC CTA CG -3'<br>5'-CAC CCA GGT CAA ACA CCA G -3'
hGAPDH	5'-ATC GTG GGG CGC CCC AGG CAC -3'<br>5'-CTC CTT AAT GTC ACG CAC GAT TTC -3'
