# Supplementary material for: Abrogation of store-operated Ca2+ entry protects against crystal-induced ER stress in human proximal tubular cells
Source: Cell Death Discov. 2019 Aug 5;5:124. doi: 10.1038/s41420-019-0203-5 (PMC6680047; doi:10.1038/s41420-019-0203-5)
Supplement: Supplementary file 2 — Supplemental Material File #1 [file 41420_2019_203_MOESM2_ESM.docx]

**SUPPLEMENTAL MATERIAL**

Supplemental Figure 1. (A) Differential crystal staining of internalized crystals. Control (non-crystal), CaP, CaOx, and CaP+CaOx (mixed) crystals were internalized in HK2 cells and stained with alizarin red pH 6.8 (Control, CaOx and Mixed) or alizarin red pH 4.3 (CaP) and representative images (3 experiments) were obtained. Scale bar = 50 µm. (B) Primer Table. Primers used to amplify mRNAs encoding human genes based on published GenBank sense and antisense sequences.
